# Tropical deforestation induces thresholds of reproductive viability and habitat suitability in Earth’s largest eagles

**DOI:** 10.1038/s41598-021-92372-z

**Published:** 2021-06-30

**Authors:** Everton B. P. Miranda, Carlos A. Peres, Vítor Carvalho-Rocha, Bruna V. Miguel, Nickolas Lormand, Niki Huizinga, Charles A. Munn, Thiago B. F. Semedo, Tiago V. Ferreira, João B. Pinho, Vítor Q. Piacentini, Miguel Â. Marini, Colleen T. Downs

**Affiliations:** 1 Centre for Functional Biodiversity, School of Life Sciences, University of KwaZulu-Natal, P/Bag X01, Pietermaritzburg, 3209 South Africa; 2 School of Environmental Sciences, University of East Anglia, Norwich, NR47TJ UK; 3 Instituto Juruá, Rua Belo Horizonte, 19, Manaus, Brazil; 4 Departamento de Ecologia e Zoologia, Universidade Federal de Santa Catarina, Florianópolis, Santa Catarina, 88040-900 Brazil; 5 Medicina Veterinária, Universidade Federal de Mato Grosso, Sinop, 78550-728 Brazil; 6 New Mexico Highlands University, Las Vegas, NM 87701 USA; 7 HAS University, 90108, Venlo, 5200 MA The Netherlands; 8 SouthWild, Mato Grosso, Várzea Grande, 78125-048 Brazil; 9 Instituto Nacional de Pesquisa do Pantanal (INPP), Museu Paraense Emílio Goeldi (MPEG) - Programa de Capacitação Institucional, Cuiabá, Mato Grosso 78735-901 Brazil; 10 Instituto de Biociências, Programa de Pós-Graduação em Ecologia e Conservação da Biodiversidade, Universidade Federal de Mato Grosso, Cuiabá, Mato Grosso 78735-901 Brazil; 11 Departamento de Biologia e Zoologia, Instituto de Biociências, Programa de Pós-Graduação em Zoologia, Universidade Federal de Mato Grosso, Cuiabá, Mato Grosso 78735-901 Brazil; 12 Departamento de Zoologia, IB, Universidade de Brasília, Brasília, Distrito Federal, 70910-900 Brazil

**Keywords:** Ecology, Environmental sciences, Ecology, Conservation biology

## Abstract

Apex predators are threatened globally, and their local extinctions are often driven by failures in sustaining prey acquisition under contexts of severe prey scarcity. The harpy eagle *Harpia harpyja* is Earth’s largest eagle and the apex aerial predator of Amazonian forests, but no previous study has examined the impact of forest loss on their feeding ecology. We monitored 16 active harpy eagle nests embedded within landscapes that had experienced 0 to 85% of forest loss, and identified 306 captured prey items. Harpy eagles could not switch to open-habitat prey in deforested habitats, and retained a diet based on canopy vertebrates even in deforested landscapes. Feeding rates decreased with forest loss, with three fledged individuals dying of starvation in landscapes that succumbed to 50–70% deforestation. Because landscapes deforested by > 70% supported no nests, and eaglets could not be provisioned to independence within landscapes > 50% forest loss, we established a 50% forest cover threshold for the reproductive viability of harpy eagle pairs. Our scaling-up estimate indicates that 35% of the entire 428,800-km^2^ Amazonian ‘Arc of Deforestation’ study region cannot support breeding harpy eagle populations. Our results suggest that restoring harpy eagle population viability within highly fragmented forest landscapes critically depends on decisive forest conservation action.

## Introduction

Securing an adequate food supply is a biological imperative that drives fundamental elements of a species biology, including mating^1^, risk assessment^2^, survivorship^3^ and geographic range boundaries^4^. Consequently, feeding ecology studies have become central to population ecology^3^, and have shown how consumer density is linked to food abundance^5^ and why some species can persist in human-dominated landscapes^6–8^, while others cannot^9–11^. Apex predators are particularly sensitive to changes in the spectrum and availability of food because of their high metabolic requirements^12^. They depend on stable and secure prey populations to fuel their relatively high daily survival and breeding requirements^13^.

Anthropogenic habitat degradation and prey scarcity induced by poaching forces predators to adapt to feeding on alternative prey species to meet their basic metabolic needs. There are several examples of prey switching as a consequence, including jaguars (*Panthera onca*) that feed mostly on armadillos (Cingulata) in semi-defaunated habitats^14^, and golden eagles (*Aquila chrysaetus*) that feed extensively on mesopredators at sites lacking their usual lagomorph prey^15^. Some natural predators may switch to domestic livestock^8,16–18^, but once exacerbated by habitat loss, these human-wildlife conflicts often drive the landscape-scale extirpation of apex predators^9,10^. Predators may adjust to these changes and thrive in anthropogenic landscapes dominated by an agricultural matrix as suitable prey remains abundant, and humans show tolerance^19–21^. Consequently, the threshold of minimum food availability—which can be used to predict the population viability and persistence of apex predators and their coexistence with humans—has been extensively investigated in degraded landscapes^22–24^.

**Figure 1:**
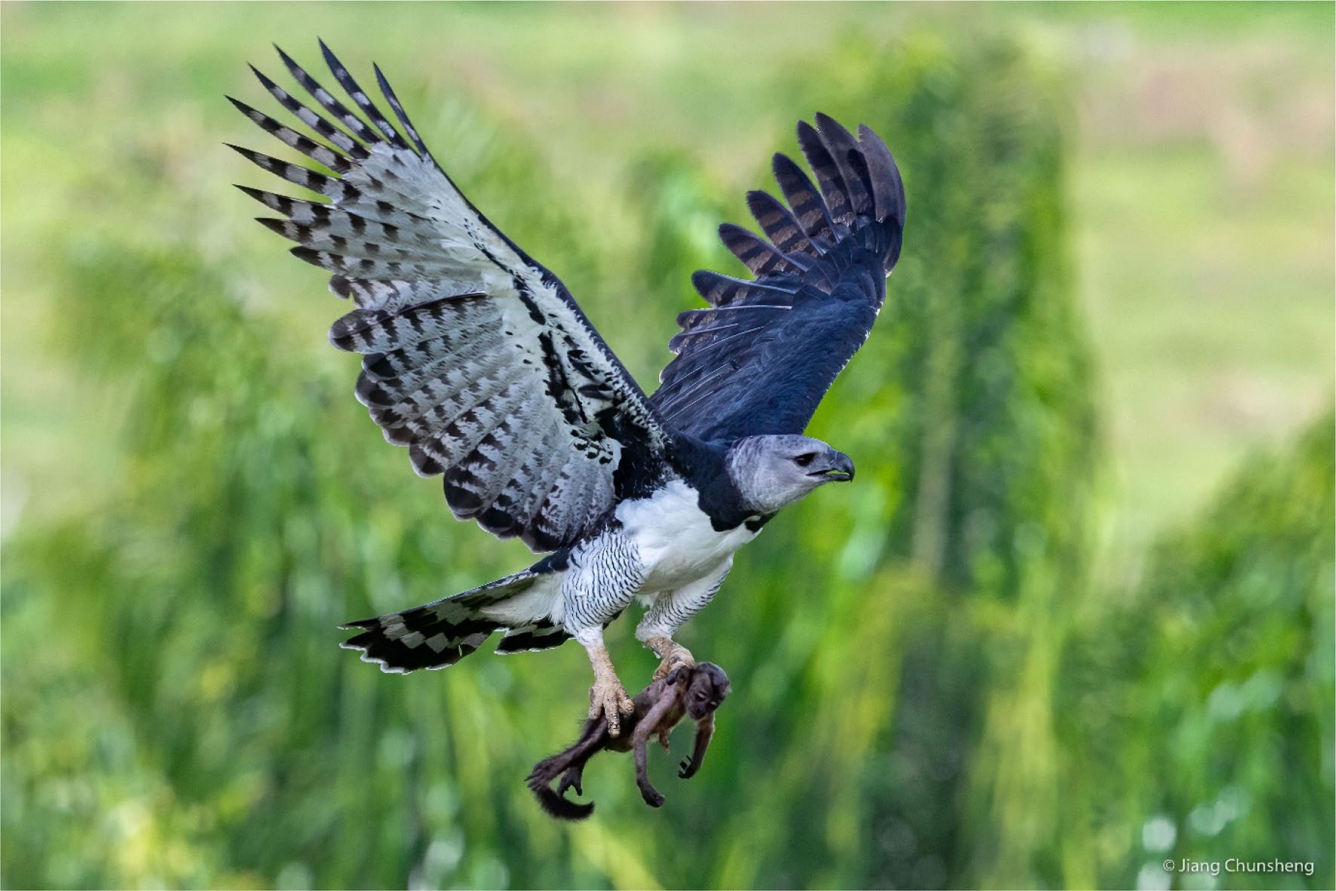
Harpy eagle (*Harpia harpyja*) arriving at a nest with a brown capuchin monkey (photo: Jiang Chunsheng).

The harpy eagle (*Harpia harpyja*; Fig. 1) is an apex predator that feeds primarily on forest canopy vertebrates^25,26^. As one of the Earth’s largest eagles (males and females averaging 5.9 and 7.3 kg, respectively), they have stringent ecological requirements^27^ that include ~ 800 g of food per day for adults^28^ and emergent nest trees taller than 40–45 m^29^. Harpy eagles have a 30–36-month-long breeding cycle, lay two eggs but fledge only a single eaglet^30–33^. Harpy eagles are typically very long-lived; a 54 years-old wild-caught adult individual was still alive with the publication of the latest studbook^34^. They usually show strict nest site fidelity, breeding in the same specific T-shaped emergent nest tree for several consecutive decades^35,36^. These trees are typically of commercial interest to the logging industry^29,37^. Studies of their breeding biology are still considered a high research priority^33^. Their global distribution has contracted by 41% since the nineteenth century, and currently, 93% of their distribution range is within Amazonian Forests, their last stronghold^10^. Leading causes explaining their rapid decline are habitat loss^10^, and shooting by settlers and, to a lesser extent, reprisal for killing livestock^38,39^.

Harpy eagles exert strong top-down control on their prey populations^28,40^. The literature widely describes sloths as the main prey taxon, followed by mid-sized primates^26^. Large reptiles (notably green iguanas, *Iguana iguana*) and large birds (such as guans and curassows; Cracidae) are less important prey species for harpy eagles^26^. Terrestrial ungulates such as brocket-deer (*Mazama* spp.) and peccaries (Tayassuidae) are even less frequent in their diet^25^. Studying how landscape degradation affects the feeding ecology of harpy eagles can shed light on how they adjust their diet with habitat loss. While dietary profiles are the main aspect studied in harpy eagle ecology, with over 1000 prey records available in the literature^25^, there is no information on the impacts of landscape-scale primary habitat conversion on the feeding ecology of harpy eagles. Yet ecological limitations such as habitat loss and prey scarcity are key to understanding their extirpation thresholds in human-modified landscapes.

To understand the impacts of forest loss on harpy eagle feeding ecology, our aims here were fourfold. Firstly, we examined the effects of harpy eagle sexual dimorphism on their feeding ecology. We predicted that larger-bodied females would prey on larger-bodied and less diverse prey species than smaller males. Secondly, we tested the effects of landscape degradation on predation rates of non-forest and disturbance-tolerant prey species. We predicted that pairs nesting in highly deforested sites would prey on these species, and sustain a more diverse diet. Thirdly, we investigated the effects of landscape degradation on numerical and biomass rates of prey delivery to eaglets. We predicted that eaglets would face lower prey delivery rates at longer intervals in degraded landscapes, resulting in lower prey biomass acquisition. Lastly, we scaled up our estimates of the deforestation tolerance threshold of harpy eagles and associated prey scarcity to a much larger human-modified region of the Amazon.

By exploring the above, we were able to estimate the consequences of extensive tropical deforestation on the persistence of breeding pairs of harpy eagles. Finding and capturing sufficient prey is the key issue for the persistence of such an apex predator. Therefore, their population viability in human-modified landscapes depends on their tolerance threshold to food stress, which is presently unknown.

## Results

### Prey profiles

We recognised a total of 306 prey items from harpy eagle nests, 253 of which from camera-traps and 53 from bone material. From a total of 279 prey deliveries recorded by camera-traps, 253 (89.7%) were identified to the level of species or genus across 16 different harpy eagle nests. We complemented camera-trapping data with analysis of skeletal detritus collected both within and underneath nests. We found bones of 76 different individual prey, of which 53 (69.7%) could be identified. We recorded 92 and 75 prey deliveries to nests by female and male eagles, respectively (♀♀ 34.5 vs. ♂♂ 27.2%). An additional 74 (26.9%) prey items observed on nests could not be unambiguously linked to delivery by an adult eagle. These additional prey of unknown origin either resulted from the eaglet hunting its own prey, or camera traps failing to record the adult’s arrival. For the remaining 34 prey deliveries (12.3%), we could not identify the sex of the adult because its talons were not visible in the camera trap images. All prey deliveries, combined with bone identification analysis, yielded 306 prey items representing 37 vertebrate species (Table 1). These were dominated by arboreal mammals, with three top-ranking species representing 49.7% of all deliveries and 50.0% of the overall prey biomass: two-toed sloths (*Choloepus didactylus*, 23.9%), brown capuchin monkeys (*Sapajus apella*, 18.3%) and grey woolly monkeys (*Lagothrix cana*, 7.5%).

**Table 1:** Species composition of harpy eagle prey items recorded in this study from both skeletal material and camera-trapping data.

Species	%	Camera	Bones	Biomass	M	F
Two-toed sloth *Choloepus hoffmanni*	23.9	47	26	18.8	10.14	25.00
Capuchin monkey *Sapajus apella*	18.3	52	4	15.5	31.88	19.05
Woolly monkey *Lagothrix cana*	7.5	21	2	15.7	1.45	15.48
Brazilian porcupine *Coendou prehensilis*	6.2	17	2	7.3	4.35	7.14
Spider monkey *Ateles chamek*	5.6	13	4	13.8	0.00	5.95
Black vulture *Coragyps atratus*	3.3	3	7	0.7	1.45	0.00
Coati *Nasua nasua*	2.9	9	0	3.1	4.35	0.00
Red-nosed saki monkey *Chiropotes albinasus*	2.6	8	0	2.8	1.45	4.76
Lesser anteater *Tamandua tetradactyla*	2.6	7	1	3.9	2.90	3.57
Opossum *Didelphis* sp.	2.9	7	2	0.9	0.00	2.38
Dusky titi monkey *Plecturocebus* sp.	2.3	6	1	1.0	1.45	0.00
Golden-and-blue macaw *Ara ararauna*	2.3	6	1	0.6	7.25	0.00
Peccaries *Tayassuidae* spp.	2.0	6	0	5.2	1.45	1.19
Squirrel monkey *Saimiri ustus*	2.3	7	0	0.9	4.35	2.38
Kinkajou *Potos flavus*	1.6	5	0	1.0	1.45	1.19
Woolly opossum *Caluromys* sp.	2.0	6	0	0.2	7.25	0.00
Crested curassow *Crax fasciolata*	1.0	3	0	1.1	2.90	2.38
Howler monkey *Alouatta* sp.	1.0	3	0	2.4	4.35	0.00
Parrot *Amazona* sp.	1.0	2	1	0.2	1.45	0.00
Guan *Penelope jacquacu*	1.0	3	0	0.6	1.45	1.19
Pygmy anteater *Cyclopes xinguensis*	1.0	3	0	0.1	0.00	3.57
Marmosets *Mico* sp.	1.0	3	0	0.1		
Common armadillo *Dasypus septemcinctus*	1.3	3	1	0.3	0.00	1.19
Trumpeter *Psophia viridis*	0.3	1	0	0.2	1.45	0.00
Dwarf porcupine *Coendou roosmalenorum*	0.3	1	0	0.1	0.00	1.19
Black porcupine *Coendou nycthemera*	0.3	1	0	0.1	1.45	0.00
Red-and-green macaw *Ara chloropterus*	0.3	1	0	0.3	1.45	0.00
Night monkey *Aotus azarae*	0.3	1	0	0.1	1.45	0.00
Brocket deer *Mazama* sp.	0.3	1	0	0.4		
Cocoi heron *Ardea cocoi*	0.3	1	0	0.5		
Razor-billed curassow *Pauxi tuberosa*	0.3	1	0	0.4	0.00	1.19
Green iguana *Iguana iguana*	0.3	1	0	0.3		
Tayra *Eira Barbara*	0.3	1	0	0.4	0.00	1.19
Crab-eating fox *Cerdocyon thous*	0.3	1	0	0.2	1.45	0.00
Channel-billed toucan *Ramphastos vitellinus*	0.3	1	0	0.0	1.45	0.00
Green ibis *Mesembrinibis cayennensis*	0.3	1	0	0.1		
Agouti *Dasyprocta* sp.	0.3	0	1	0.4		
Unidentified vertebrates		26	23			
Total		355				

One peccary could be identified as *Tayassu pecari*, all others were too young to be distinguished as either *Tayassu pecari* or *Pecari tajacu*. One woolly opossum was a *Caluromys lanatus*, all others could not be identified below genus. Data explicitly associated with either harpy eagle male (M) or female providers (F) at 16 nests in Southern Amazonia are from camera-trapping only. Proportional contributions in terms of prey biomass and the two sexes are expressed in in percentages.

**Figure 2:**
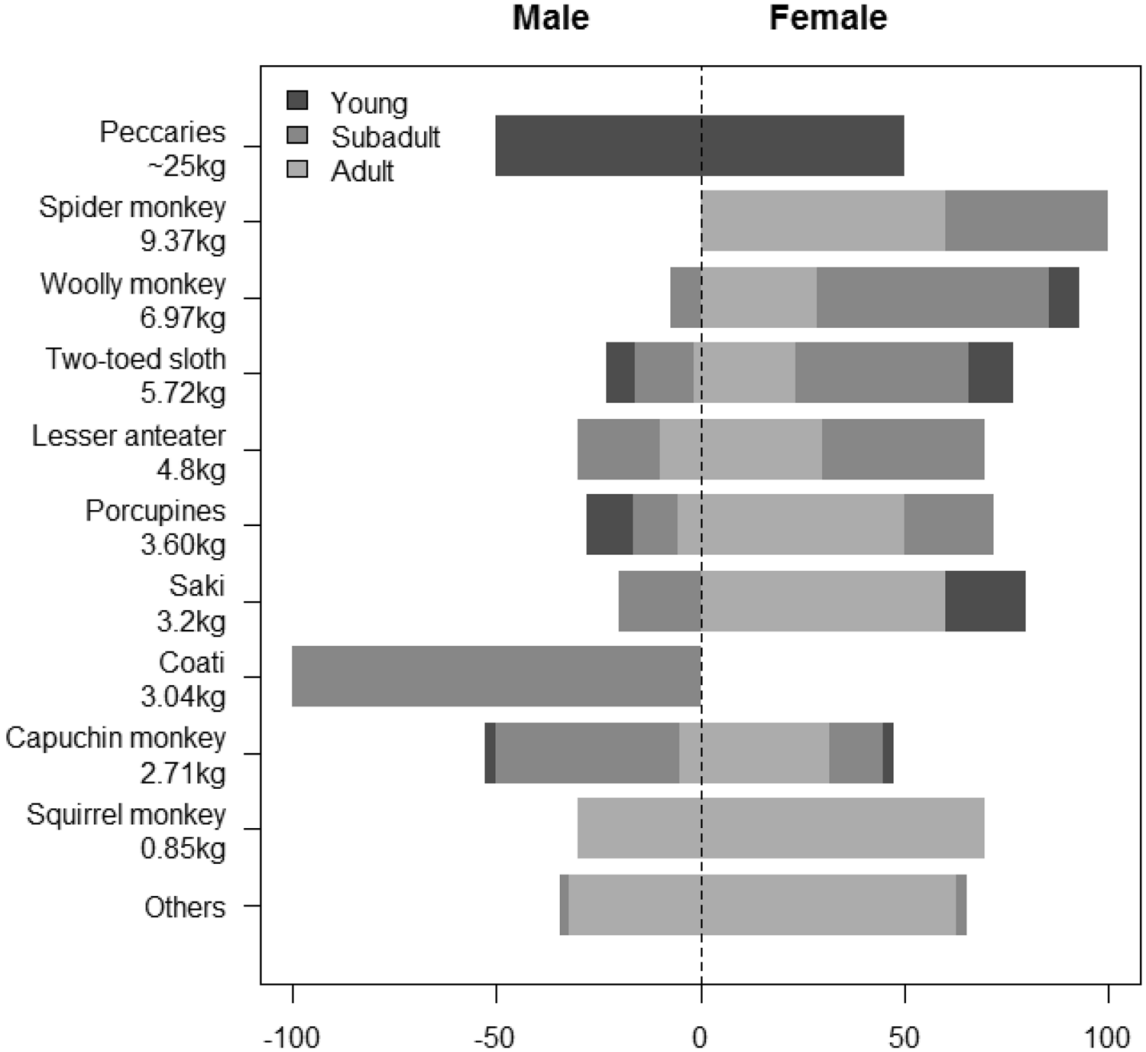
Prey age classes consumed by either male and female harpy eagles. The 10 most important prey species (representing > 80% of the total) are ordered top to bottom by adult body mass. Prey age class (stacked horizontal bars) are indicated according to the legend. Male harpy eagles are less likely to pursue adult prey, particularly in large-bodied prey species.

**Figure 3:**
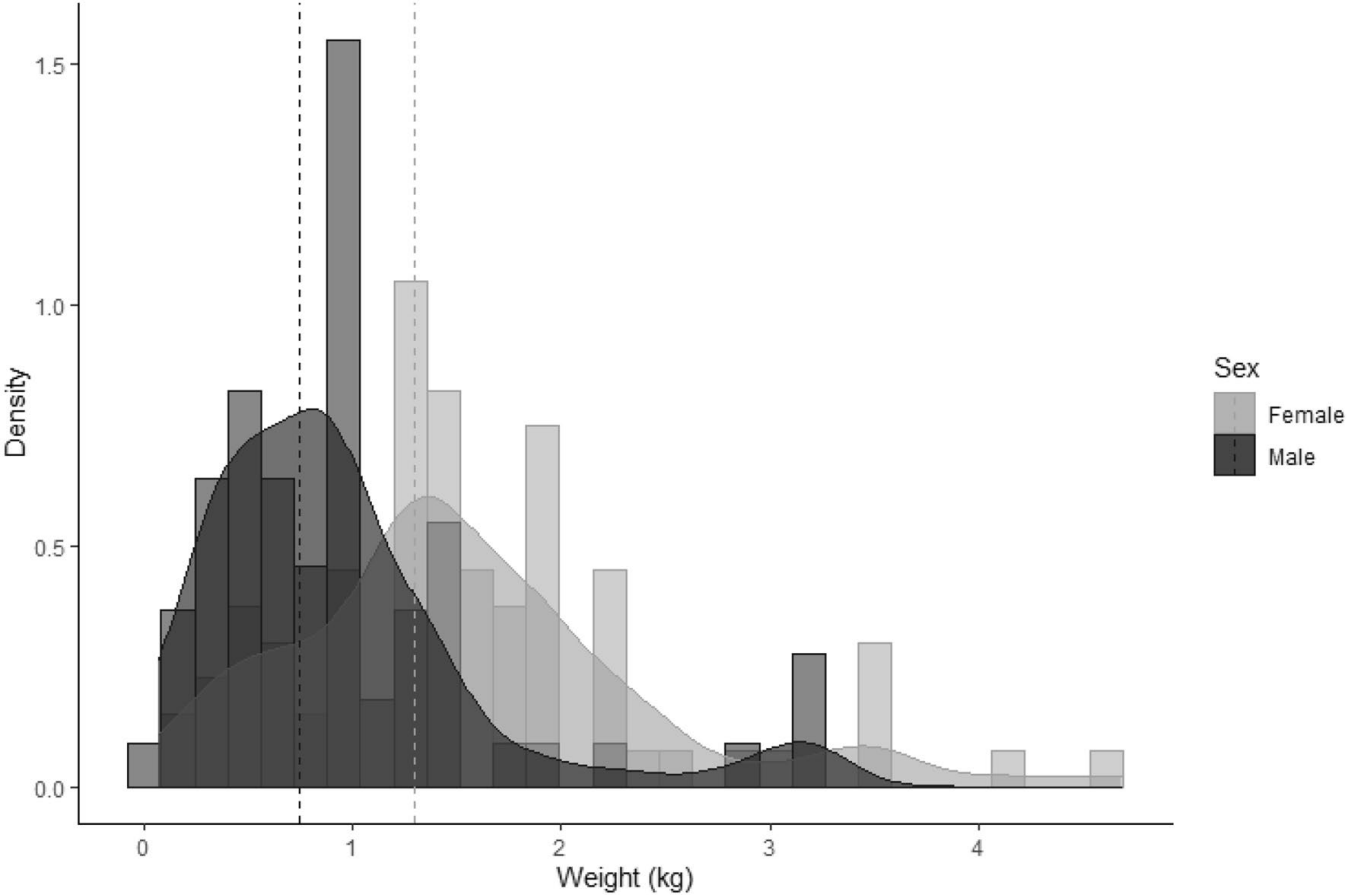
Prey body mass brought into nests by either male (n = 75) or female (n = 92) harpy eagles. Male prey averaged 0.74 kg, while female prey was 57.3% heavier, averaging 1.29 kg (hatched lines). The largest individual prey items brought into a nest were a ~ 4.7 kg lower body of a spider monkey killed by a female, and a ~ 3.2 kg lower body of a howler monkey killed by a male.

Females and males preyed on proportionally similar arboreal prey (♀♀ 75.8 vs. ♂♂ 70.6%), whose difference is not statistically significant (null model, *P* = 0.756). The same applied to both scansorial (♀♀ 17.5 vs. ♂♂ 16.0%, null model, *P* = 0.611) and terrestrial prey (♀♀ 4.4 vs. ♂♂ 6.6%, null model *P* = 0.283). Females preyed primarily on adult prey, while the smaller males preferred juveniles and subadults (Fig. 2), which led to a significant difference in prey body mass between the sexes. Male prey averaged 0.74 ± 0.69 kg (geometric mean ± SD), while female prey was 57.3% heavier, averaging 1.29 ± 0.88 kg (null model, *P* < 0.001, Fig. 3). The largest individual prey brought to a nest by any female was a ~ 4.7-kg, lower body section of an adult spider monkey, while for any male, this was a ~ 3.2 kg lower body quarters of a howler monkey. One adult whose sex could not be identified simultaneously carried into a nest the lower body of a common opossum (*Didelphis* spp.) and the lower body of a juvenile spider monkey (totalling ~ 3.6 kg). Levin’s niche width analysis yielded similar values for the sexes: 7.20 for females and 7.42 for males (n = 84 ♀♀, 69 ♂♂). Following bootstrapping based on a null model, however, the niche width fell to 2.40 for females and 4.62 for males, which was significantly different (*P* < 0.001). Photographic problems induced by mismatched fields of view or malfunctioning camera traps rendered 29% of all cameras inoperative (see Supplementary Information Table S1).

### Nest density and buffer definition

**Figure 4:**
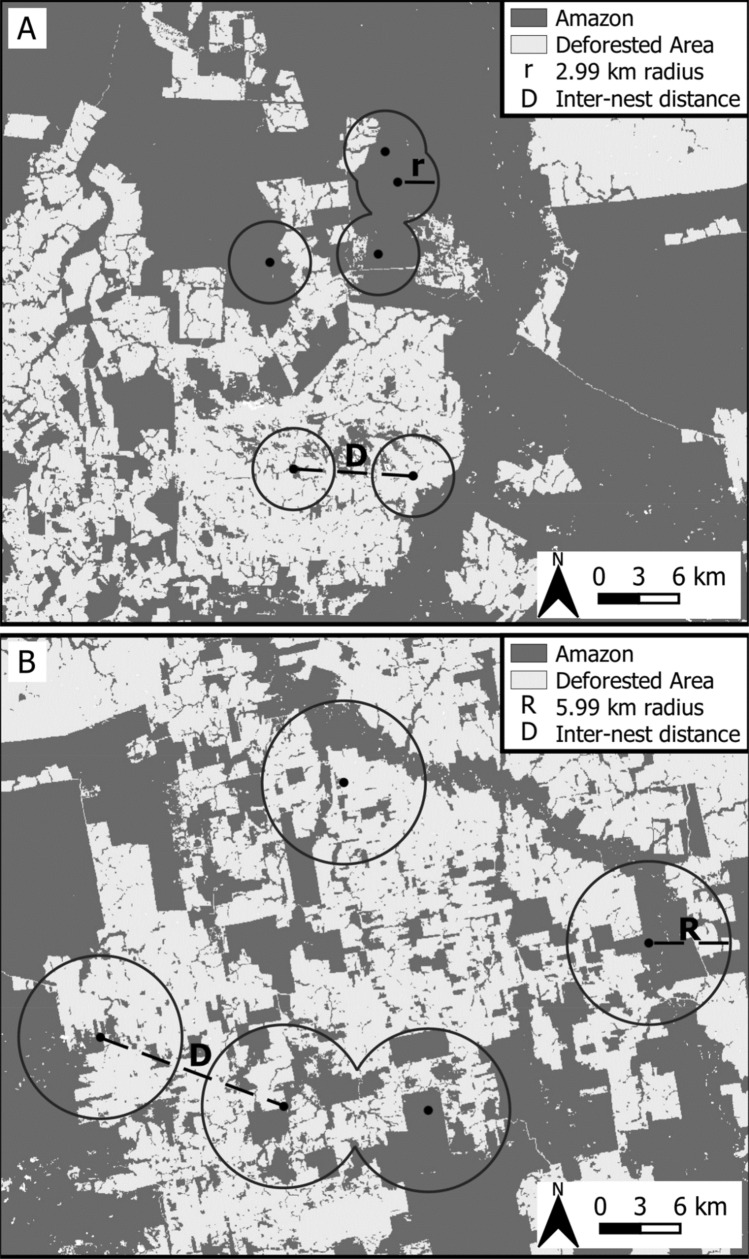
Examples of the spatial distribution of active breeding nests. Nest density was calculated by the Maximum Packed Nest Density (MPND) method. Distances to the nearest neighbouring nest were 2.99 km to 5.90 km depending on the nest cluster identity, which we used to calculate the 3-km and 6-km landscape buffers. The two southernmost nests in cluster A are the ones excluded for sitting in riparian forests (QGIS V.3.16; https://www.qgis.org).

Using the maximum packed nest density method, the average distance to the nearest neighbouring harpy eagle nest within a nest cluster was 2.99 km (Cluster A, n = 6, SD = 1.52) and 5.90 km (Cluster B, n = 5, SD = 0.89). A total of 124 km^2^ of the 195 km^2^ previously forested area around Cluster A still retained forest cover, while forest cover spanned 254 km^2^ of the 633 km^2^ around Cluster B. These data translated into a nest density of 1.97–4.84 nests/100 km^2^ of forest habitat, or 0.79–3.07 nests/100 km^2^ if we also include deforested areas (Fig. 4). We thus used both the 3-km and 6-km radii as buffers for landscape analyses. A rough approximation between these (4 km, or 50 km^2^) was used as the breeding territory size (consistent with the literature^41^), and therefore used as the hexagonal cell size for the regional-scale analysis of habitat loss.

### Prey profile responses to deforestation

**Figure 5:**
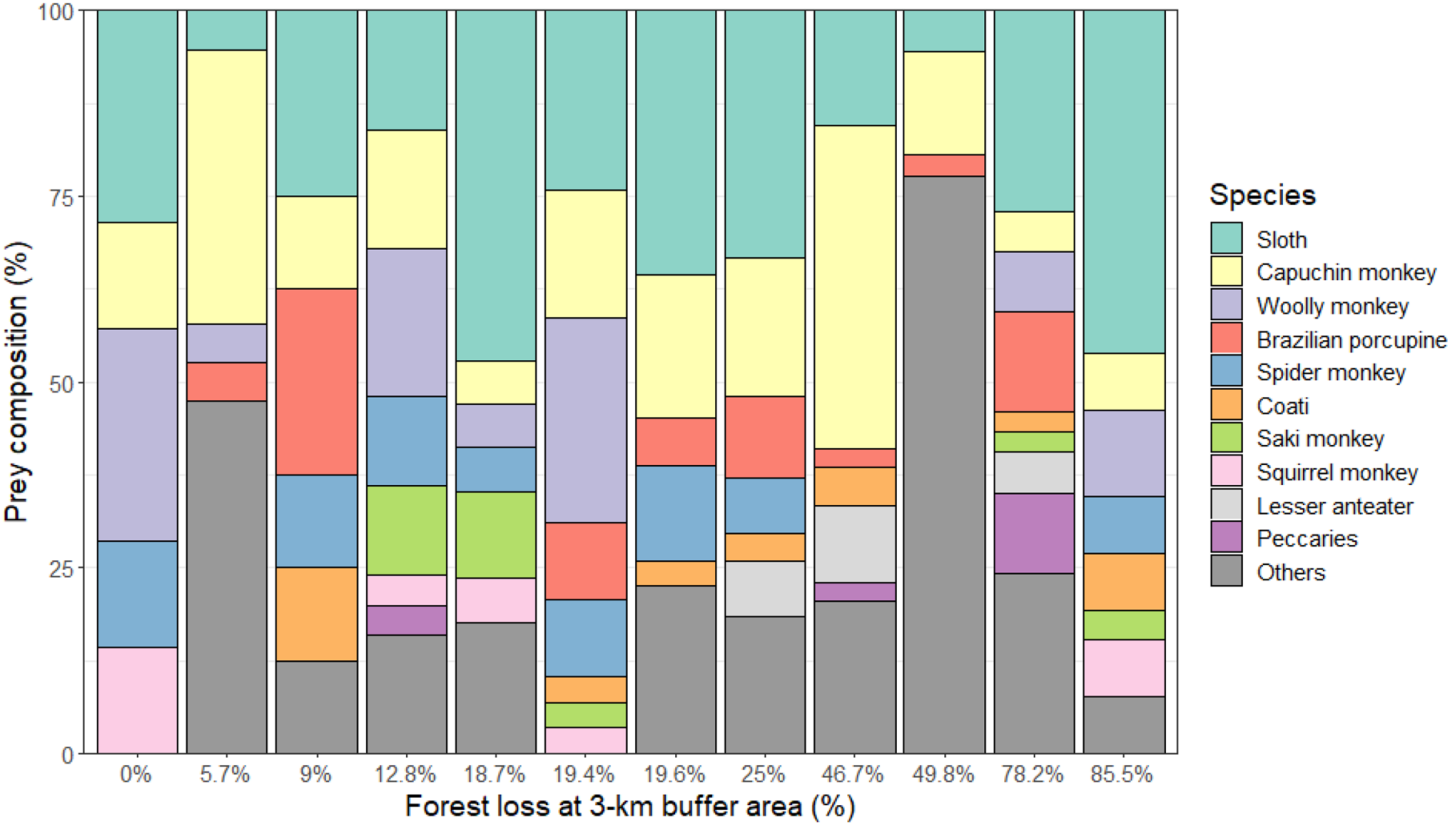
Species composition of prey items delivered to active harpy eagle nests. Main prey species are ordered in terms of overall abundance. Each column represents a nest, ordered by the landscape context from the lowest to the highest amount of forest loss within a 3-km buffer area around each nest site. The harpy eagle prey base consists of arboreal forest vertebrate species even when nests are situated in highly deforested landscapes.

**Figure 6:**
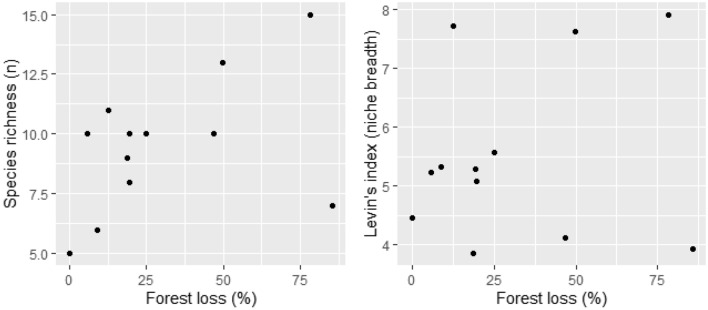
Effects of forest loss (3-km buffer) on the predation ecology of harpy eagles. Eagles nesting in highly deforested landscapes exhibited levels of prey diversity and dietary niche breadth that were similar to those in more relatively intact landscapes, with sloths and primates as the main prey.

Contrary to our expectations, the level of deforestation did not significantly alter the prey species composition for harpy eagles (Fig. 5), nor did distance to cattle pastures (range: 0–6300 m, X̅ = 1159 ± 1703 m, n = 14 nests) or amount of forest cover within a 3-km radius (range: 14–100%, X̅ = 69 ± 28%; NMDS, 999 permutations, stress = 0.1335, *P* = 0.332 for forest loss and 0.079 for distance to pasture, n = 14 nests). Eagles continued to capture sloths and primates as their main prey, even in nests sited in severely deforested areas. The degree of forest loss did not affect Levin’s niche breadth index nor prey species richness (GLM, residual deviance: 152.59 on 10 DF, *P* = 0.23, n = 12 nests), showing that harpy eagles relied primarily on large arboreal prey and barely switched to open-country terrestrial vertebrates even in highly-deforested landscapes (Fig. 6).

### Effects of deforestation on prey delivery and biomass

**Figure 7:**
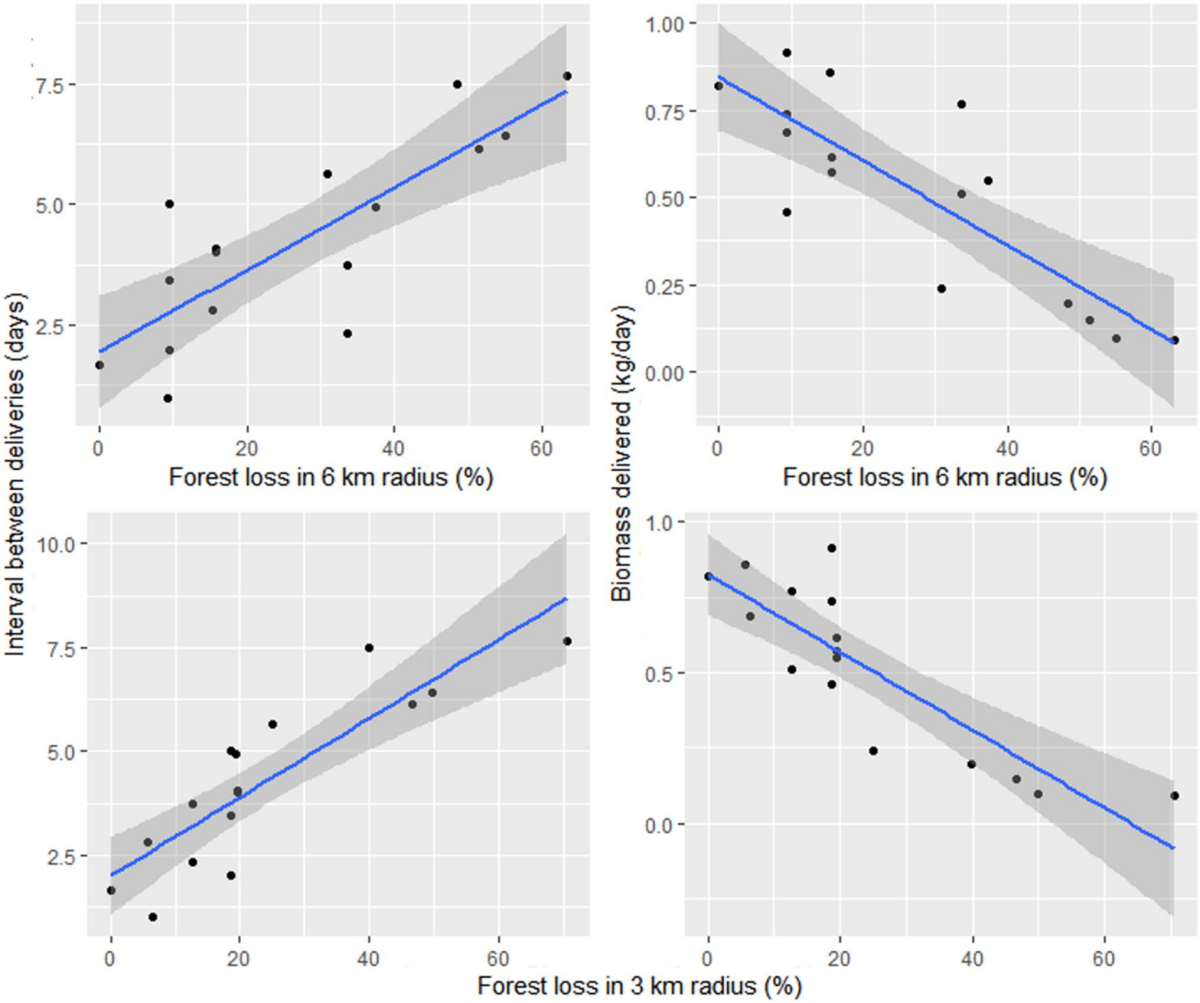
Effects of forest habitat loss on the feeding ecology of harpy eagles, as depicted by 16 nest-phases. Habitat loss reduced the frequency of prey delivery (right panels) as well as the prey biomass delivered to the nest (left panels). These slopes were significant in all cases.

Data from 16 nest-phases (12 nests) and 189 prey delivery events showed that habitat loss induced significant food stress on nesting harpy eagles (Fig. 7). For these analyses, we excluded the two nests that were located in otherwise deforested riparian forest remnants, which are clear outliers because they experienced abnormally high prey biomass delivery rates, which was equivalent to breeding territories in entirely forested landscapes. Prey delivery intervals were on average 4.20 ± 1.97 days (n = 16 nest-phases). Prey biomass delivery rates averaged 0.37 ± 0.27 kg/day (range: 0.10–0.65 kg/day, n = 16 nest-phases). Details on prey body parts brought to the nests are shown in Table 2. The nesting phase had a negative effect on prey delivery rates, with longer time intervals recorded for older eaglets. Forest habitat loss resulted in an increase in the number of days between consecutive prey deliveries considering both the 3-km (GLMM, *F*_2,11_ = 102.06, *P* < 0.001 for forest cover; *F*_2,11_ = 795.01, *P* < 0.001 for nesting phase, n = 16 nest-phases) and the 6-km buffers (GLMM, *F*_2,11_ = 35.74, *P* < 0.001 for forest cover; *F*_2,11_ = 780.82, *P* < 0.001 for nesting phase, n = 16 nest-phases). Habitat loss resulted in lower delivery rates in terms of prey biomass, which was significant for both the 3-km (GLMM, *F*_2,11_ = 34.80, *P* < 0.001, n = 16 nest-phases) and 6-km buffers (GLMM, *F*_2,11_ = 27.29, *P* < 0.001, n = 16 nest-phases). For instance, prey delivery to nests within entirely forested landscape contexts was on average 0.69 kg/day, but this declined to only 0.11 kg/day for nests in areas deforested by 50% or more. There were no effects of the nesting phase (nestling, fledged and dependent juvenile) on the prey biomass delivered.

**Table 2:** Prey body part brought into the nest for the 10 most common prey species.

Prey species	N	Full body	Beheaded	Upper body	Member missing	Single member	Lower body	Tail
Capuchin monkeys (*Sapajus apella*)	46	6.5	2.2	0.0	8.7	2.2	80.4	0.0
Two-toed sloth (*Choloepus hoffmanni*)	41	2.4	0.0	85.4	7.3	0.0	2.4	0.0
Woolly monkey (*Lagothrix cana*)	20	5.0	5.0	0.0	0.0	0.0	80.0	10.0
Porcupines (*Coendou* spp.)	17	0.0	0.0	0.0	5.9	0.0	58.8	35.3
Spider monkey (*Ateles chamek*)	9	0.0	0.0	0.0	0.0	0.0	77.8	22.2
Coati (*Nasua nasua*)	9	22.2	11.1	0.0	0.0	0.0	66.7	0.0
Saki monkey (*Chiropotes albinasus*)	8	11.1	0.0	0.0	0.0	0.0	77.8	0.0
Squirrel monkey (*Saimiri ustus*)	7	28.6	0.0	0.0	0.0	0.0	71.4	0.0
Lesser anteater (*Tamandua tetradactyla*)	6	33.3	0.0	16.7	0.0	0.0	33.3	16.7
Peccaries (*Tayassuidae* spp.)	6	33.3	0.0	0.0	0.0	16.7	50.0	0.0

All prey items, other than squirrel monkeys, brought into the nest as a whole carcass (‘Full body’ column) were either infants or juveniles. This table represents species accounting for > 80% of all prey items. For most prey, only the lower body carcass was brought into the nest, except for sloths, for which harpy eagles more frequently bring the upper body. All prey species are represented in percentages of individuals (shown in column N).

### Food stress threshold

**Figure 8:**
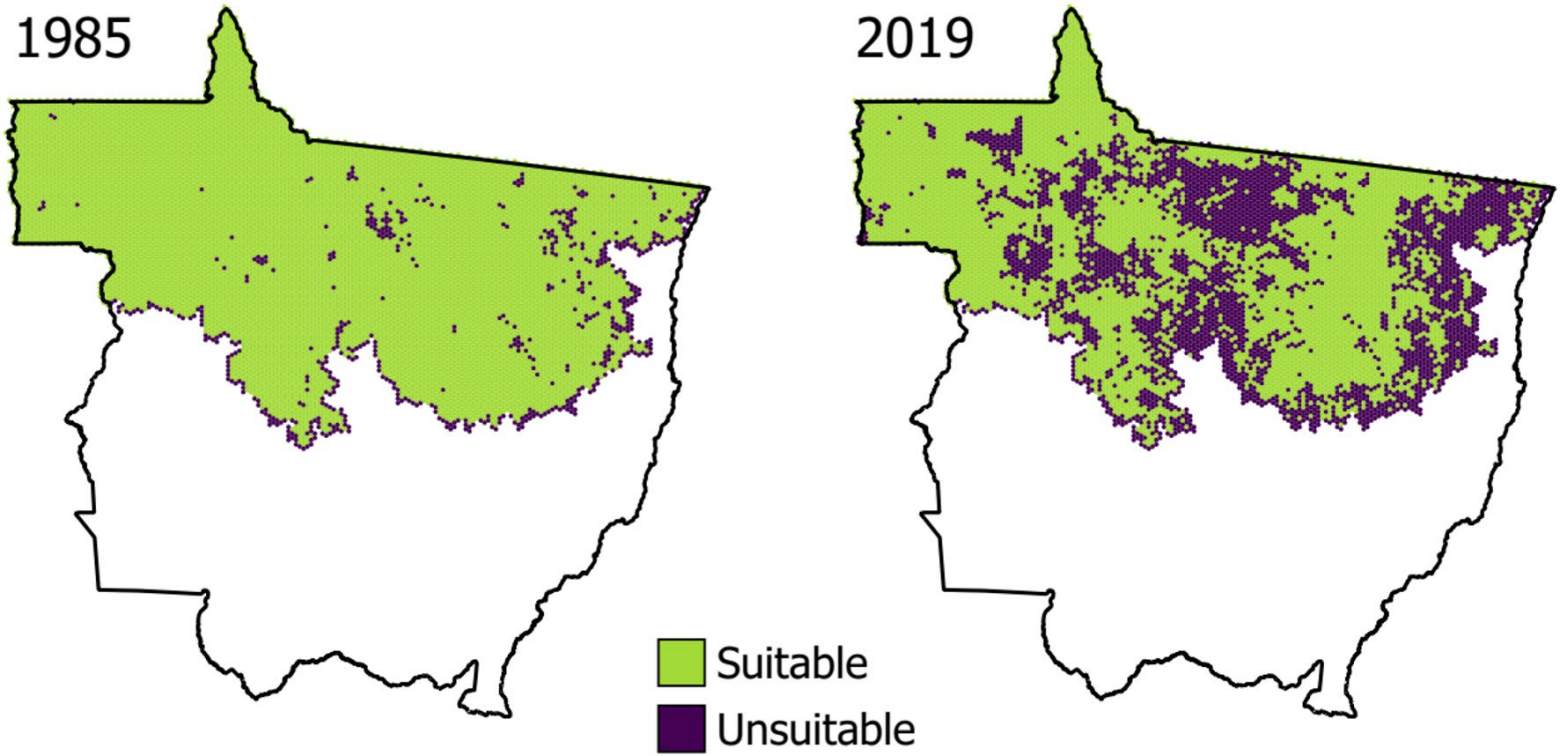
Comparison of cells in northern Mato Grosso containing less than 50% of forest (purple cells indicate unsuitable habitat) along the time series examined in this study, from 1985 (left) to 2019 (right) in a region that was once entirely covered by Amazonian forest. No harpy eagle nests has ever been found in landscapes with less than 30% of forest cover, and pairs attending nests in areas surrounded by 30–50% forest cover where unable to feed nestlings/fledglings into the dependent juvenile phase (see “Results” section). We therefore consider 50% to be the harpy eagle habitat loss threshold in terms of reproductive viability, at least in terms of completing a nesting cycle. We further estimate that 35% of northern Mato Grosso can no longer support reproductively viable populations of harpy eagles (QGIS V.3.16; https://www.qgis.org).

Considering that we found no active nests in landscapes that had succumbed to > 70% forest loss (n = 34, excluding nests in riparian forest remnants), we determined an approximate threshold above which harpy eagles can no longer tolerate landscape-scale deforestation. Yet harpy eagle pairs nesting within landscapes containing 50–70% forest cover were unable to feed fledged eaglets up to the stage of dependent juveniles. We witnessed three fledged eaglets dying of starvation at three nests surrounded by 63.3%, 54.3% and 53.0% of deforestation at 3-km buffer. We, therefore, consider levels of forest habitat loss higher than 50% as prohibitive for successful nestling rearing in harpy eagles. Under this assumption—and considering the 50-km^2^ hexagons—we estimate that forest habitat loss in the southern Amazon has extirpated harpy eagle populations from 35% of their original distribution throughout northern Mato Grosso (Fig. 8). We further estimate that this represents a habitat-induced metapopulation decline of ~ 3256 breeding pairs of harpy eagles. This is particularly concerning given that 28.7% of this entire region has been deforested in the last 35 years, with only 64.9% of forest cover currently remaining.

## Discussion

Our data and analyses showed that forest loss is associated with severe reductions in prey delivery rates and prey biomass by adult harpy eagles to their eaglets; nestlings reared in highly fragmented areas received less food with longer intervals between consecutive feeding bouts. On the other hand, prey species composition changed relatively little in areas that had succumbed to varying amounts of deforestation. This provides clear evidence that harpy eagles depend heavily on forest canopy vertebrate prey. In fact, they exhibited limited hunting ability in non-forest areas, including cattle pastures, targeting few terrestrial vertebrates. These traits render them unable to both use any nest surrounded by > 70% forest loss, or feed young until they reach the dependent juvenile phase in nests located within areas deforested by 50–70%. These findings provide the basis for understanding the persistence thresholds of reproductively viable harpy eagle populations within fragmented forest landscapes.

The dietary profile of harpy eagles in northern Mato Grosso shows that sloths comprised only a quarter of all prey items, which is much lower than studies elsewhere in which sloths account for at least two-thirds of all prey^26^. Importantly, three-toed sloths (*Bradypus* spp.), which are normally the most important components of harpy eagle diets^26,42^, were largely absent in our study region. The absence of three-toed sloths in this region of southern Amazonia is confirmed by several reports of arboreal wildlife rescue operations in hydroelectric dams^43^. Differences between the age and size of prey taken by male and female harpy eagles were similar to those in previous studies. However, in terms of prey habits (terrestrial or scansorial), the differences were smaller than typically observed in unpaired harpy eagles^44^. Another surprising pattern emerging from our study area was the conspicuous absence of small livestock in harpy eagle diets. Other apex predators in the same landscape, such as jaguars, frequently prey on bovine cattle^18^. We conducted prior research in the region to understand the prevalence of retaliatory killings of harpy eagles. While the reported prevalence of livestock predation was relatively low (< 20% of 184 interviewees^38^), we expected eagles to kill small livestock, especially those nesting near homesteads. Perhaps livestock predation is primarily associated with floaters and therefore was undetected in nests where breeding pairs did virtually all the hunting.

In particular, we found that harpy eagles took a large number of relatively large-bodied prehensile-tailed (Atelinae) primates (both spider monkeys *Ateles chamek* and woolly monkeys *Lagothrix cana*). It has been suggested that, as an advantage of their large size, these relatively large-bodied neotropical primates are virtually exempt from aerial predation^45^. The general absence of previous records of harpy eagles preying on large Atelinae primates has led primatologists to consider these predation events as extremely rare. We suspect that this could be partly explained by the fact that our study area does not experience human hunting of Atelinae primates, and consequently boasts high population densities of large-bodied monkeys^43,46^. In northern Mato Grosso, hunting of canopy vertebrates is mostly limited to gamebirds such as guans and curassows^47^. This is largely attributed to cultural taboos against eating monkeys and sloths^48–50^, given that hunters in this area are typically descendants of recent migrant settlers from southern Brazil^51^. Grey woolly monkeys were frequently represented in harpy diets, particularly considering that they only occur in forests west of the Juruena River^52^, or roughly half of our study area. However, high *Ateles* and *Lagothrix* population abundance is a rare phenomenon in most parts of Amazonia^52–54^, where fragmentation and hunting often have a synergistic effect on prey density. Viable breeding populations of harpy eagles are indeed unlikely to persist in areas that are both overhunted and largely deforested, given the “double-whammy” effects of these combined stressors^53^. Our conservative estimates of landscape-scale deforestation thresholds that can be tolerated by nesting harpy eagles can therefore be considered as optimistic in most of the Amazon.

It remains unclear from our study why and how harpy eagles in other highly fragmented forest landscapes can rely so heavily on terrestrial prey such as armadillos (A. Blanco, C. Tuyama, pers. comm.). At one much more southerly harpy nest that we excluded from this study, the prey profile of adults provisioning their nest consisted almost entirely of armadillos. The two eagles that fledged on the southern outlier nest were proficient at processing armadillo carcasses. In contrast, the few armadillos recorded in this study were delivered belly-down, and the fledglings failed to turn the armadillos, which subsequently went uneaten. Prey handling skills and hunting profiles may therefore be culturally inherited and dependent on previous experience and prey availability. Another possibility is that the local abundance of armadillo prey, or the species of armadillos available, causes those differences, since naked-tailed armadillos (*Cabassous* spp.) are the vast majority of individual killed in the aforementioned nest. Predation by harpy eagles on other non-forest, open-country prey species was at best infrequent, as evidenced by the very few records of opossums, macaws, mesocarnivores and other non-forest generalist prey in their diet; harpy eagles were, therefore, unable to switch to non-forest prey species in our study area.

The Levin’s index of trophic niche breadth was much greater than the previous estimates^42^. We believe this higher trophic breadth index is associated with nest monitoring using camera-traps. Specifically, our camera-traps added 14 new species to the previous total of 102 known prey species taken by harpy eagles anywhere in their range^25^. Many prey species would have gone undetected had we relied exclusively on bones to identify prey species (Table 1), which would also induce a methodologically biased level of prey importance for sloths. Eagles carry whole small-bodied prey under specific orientation that reduces aerodynamic drag (Fig. 1). For larger prey, butchering carried out by the eagles to reduce flight drag leaves few easily identifiable body parts for most prey, as shown in Table 2. Further evidence of the importance of camera-trapping comes from our prey species composition. Had this been based exclusively on skeletal remains recovered within and underneath nests, then this would have shown only “the usual suspects” of harpy eagle prey species, with the notable exception of Atelinae primates. Therefore, we highly recommend the use of camera-traps in further feeding ecology studies of rainforest raptors. Our 89.7% prey identification rate was also higher than the 47.2% obtained using surveillance cameras^54^. However, given the several camera-trapping problems we encountered, further safeguards are necessary to reduce camera-trap failure to a bare minimum (see Supplementary Information Table S1).

The harpy eagle prey base consisted of arboreal forest vertebrate species even when nests were situated in highly deforested landscapes. As central-place foragers, breeding harpy eagles incur much greater travel costs to forage in faraway forest fragments in heavily deforested landscapes. This is probably the critical inflection point that precludes nesting in landscapes that had been deforested by > 70%, as well as failure in raising chicks in landscapes experiencing 50–70% of forest loss.

Harpy eagle prey delivery rates in terms of frequency and biomass clearly declined with forest habitat loss. On three occasions involving recently-fledged eaglets, delivery rates were so low that the fledglings died of starvation. One of those nests was monitored by camera-traps, where prey delivery intervals were consistently longer than 15 days, whereas the typical interval required to sustain recently-fledged eaglets is ~ 2.5 days. In another case in which the fledgling survived, the parents were clearly food-stressed and attempted to fall back on a diet of forest birds. That pair was responsible for all the predation events reported here on blue-and-gold macaws (*Ara ararauna*) and crested curassows (*Crax fasciolata*). Their dependent juvenile male eagle, however, quickly learned how to hunt black vultures (*Coragyps atratus*) and accounted for 9 of our 10 records of harpy predation on vultures. Hunting by recently-fledged harpy eaglets is not unheard of^55^ and is especially common in fledged males^56,57^. We emphasise that a harpy eagle requires ~ 800 g of prey each day^58^, an unfeasible target if they are forced to rely on a mixed diet of forest birds. In all other nests, adults continued to prey on sloths and primates. The impacts of sustained depletion of adjacent prey populations by a strict central-place predator hunter within ever smaller forest remnants deserves further study, as harpy eagles clearly exert strong top-down control on some prey species^28,40^.

By establishing the amount of deforestation that harpy eagles can tolerate to meet their basic ecological requirements, we can estimate the harpy eagle population size lost to cattle pastures and other non-forest land uses in the Amazonian portion of Mato Grosso. Currently, 64.9% of northern Mato Grosso still supports forest cover, which may sound encouraging in retaining harpy eagles and other forest vertebrates. Much of the remaining forest habitat, however, lies within indigenous territories^59^. The vast majority of native Amazonians kill any number of harpy eagles whenever possible to harvest the primary wing and tail feathers for headdresses and fletching arrows^60–62^, trade headdresses commercially (a practice that is illegal for tribal peoples Brazil^63^) and capture harpy eagle nestlings to keep as caged pets in Indian villages^60^ or sell them on the black market (EBPM, pers. obs.). Proactive wildlife co-management^64,65^ with indigenous peoples should be a priority, including a no-take policy for highly vulnerable species to prevent the extirpation of harpy eagles and other low-fecundity species from Indigenous Lands^66^. We support indigenous cultural practices and trust that a better wildlife policy from government bureaus and NGOs working with indigenous people will stimulate sustainable use while reinforcing the legal framework that prevents these issues.

We observed that for two harpy eagle nests sited in riparian forest remnants, prey delivery rates were high despite deforestation levels exceeding 70%. These observations are encouraging but at odds with some of our predictions since some hexagonal-cells in our maps may represent riparian corridors that remain well connected to larger forest fragments. Riparian forest remnants in northern Mato Grosso are usually too narrow, poorly connected or highly degraded to support high bird and mammal diversity^67^. They should also be linked to large fragments, or continuous forests if they are to successfully support harpy eagle reproduction and juvenile dispersal.

Over half of all riparian forest corridors in the study region are degraded to some extent^67–69^, which inhibits movements of arboreal mammals such as woolly monkeys, spider monkeys and sloths. Furthermore, selective logging is widespread in those riparian forests^68^ and removes the same set of large canopy trees that harpies require for nesting^29,68^. Additionally, the principal nest tree species used by harpy eagles^29^—the Brazil nut tree *Bertholletia excelsa*—rarely occurs within riparian sites, and is largely restricted to higher terrain^70^. One strategy that can generate resources to fund activities for greater forest connectivity, engage local communities towards conservation through economic incentives and encourage governments to create and manage corridors is ecotourism^71,72^. As nature conservation is mostly an unprofitable activity^73^, tourism is one of the few lucrative activities that can generate financing for conservation^74–76^.

Our results contribute to a greater understanding of how harpy eagle feeding ecology is impacted by anthropogenic land-use change, with prey availability frequently as the main constraint for the persistence of any apex predator. Determining the deforestation thresholds that a large forest canopy raptor can tolerate is of prime interest for conservation actions such as adding value to forest habitat through ecotourism and carrying out eagle reintroductions or translocations, which can boost populations of this species in their forest environments. Finally, it is likely that nesting harpy eagles in hyper-fragmented Neotropical forest landscapes, such as the Atlantic Forest and the rapidly expanding Amazonian Arc of Deforestation, are become increasingly isolated in relatively small fragments. These stranded eagles will likely depend on decisive “hands-on” population management interventions, which could include translocation of juveniles and food supplementation to eaglets, if they are to persist.

## Methods

### Study area

**Figure 9:**
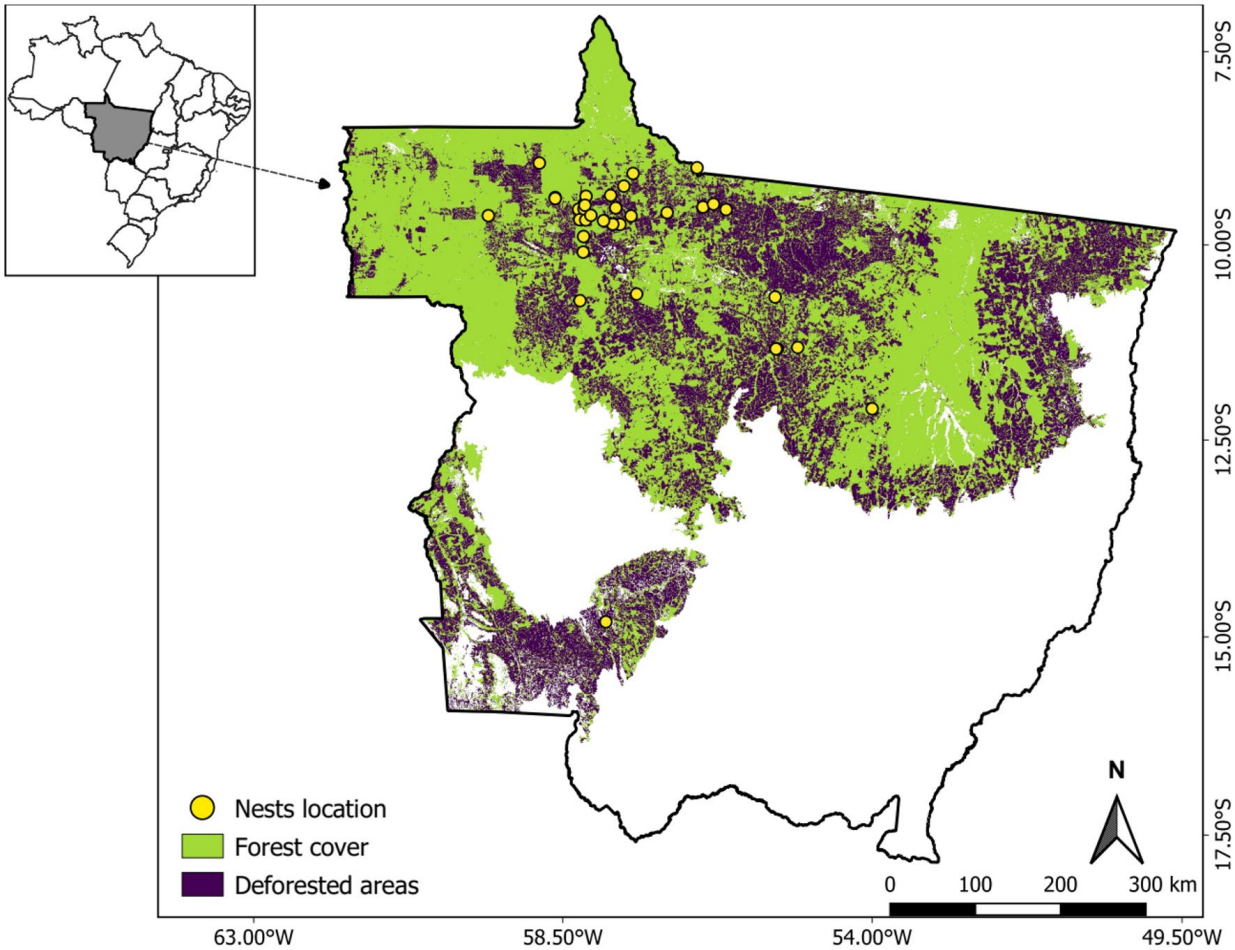
The 903,357-km^2^ state of Mato Grosso in Brazil, showing the location of harpy eagle nests monitored in this study (yellow circles). Colour-coded areas represent the Amazonian biome portion of the state. Primary forest and deforested areas (mostly cattle pastures) are shown in green and purple, respectively. Blank areas indicate non-forest vegetation. Mapping data are based on 2019 Landsat imagery geoprocessed as part of the MapBiomas project (mapbiomas.org collection 5; year 2019; QGIS V.3.16; https://www.qgis.org).

Amazonian Forests have been degraded, particularly along a large deforestation frontier known as the Arc of Deforestation^51,77^. This study was conducted in the southern portion of the Arc of Deforestation in the northern state of Mato Grosso, Brazil (Fig. 9). The main agricultural land uses in this region are cattle ranching and soybean farming^78–80^. Brazilian forestry law requires that a minimum proportion of any private landholding (varying per ecoregion, but 80% in Amazonia), as well as riparian forests along rivers and streams, should be spared from deforestation^81^. Human occupation thus generates a hyper-fragmented landscape mosaic with varying levels of habitat loss and structural connectivity^68,82^. Koeppen^83^ classifies the region’s climate as “tropical wet climate” or Amazonian (tropical monsoon) climate. Annual rainfall averages 2350 mm, and annual ambient temperature averages 24.5 °C^84^. The wet season occurs from October through to March, amounting to ~ 80% of the annual precipitation, whereas the dry season spans from April through to September. Arboreal folivores such as howler monkeys (*Alouatta* spp.) and green iguanas are locally rare in forest areas^43,46,85^, while the three-toed sloth (*Bradypus* spp.) is largely absent^43^. These prey species are important elsewhere, as reported by most previous harpy eagle dietary studies^42^. This portion of the southern Amazonia was originally inhabited by several indigenous groups^60^, but they have been largely displaced since the 1970s by southern Brazilian settlers who joined a government-sponsored transmigration program^51^. Our study region is currently dominated by cattle-ranches, with the largest forest areas remaining within National Parks, State Parks and Indigenous Lands, and most smaller forest remnants set aside as forest reserves within private landholdings^81^.

### Nest finding

We openly offered a reward amounting to ~ USD100 or BRL500 (about 50% of the Brazilian minimum monthly wage) for geographical positional information for any active harpy eagle nest reported to us. The reward was widely-publicised in posters and pamphlets that were disseminated among key groups of rural workers, indigenous people, timber industry personnel and especially Brazil-nut collectors who spend the most time in the forest. This reward payment allowed us to ‘discover’ harpy eagle nest locations unconstrained by biases related to forest location, as Brazil-nut collectors and indigenous people range widely within forest areas, while other informants were mostly urban dwellers. Once we identified a nest location, we proceeded to reach contractual agreements with the landowner. These contracts included formal agreements to ensure physical access to scientific research and low-disturbance ecotourism^86^.

Harpy eagle tourism originated as a cooperative venture between us and a private tour company that install near harpy eagle nests observation towers. Our initiative required that the company pay ~ USD20 (BRL100) per tourist per day to the landowner. In exchange, each landowner signed a legal contract stipulating several measures that prevent forest degradation^86^. Local inhabitants earned money from the project by transporting and building the towers, as well as trail-cleaning, driving, cooking for tourists and staff, and other associated logistical services^86^.

### Climbing and camera-trapping protocols

We climbed harpy eagle nests using published, raptor-specific tree-climbing protocols^87,88^. We first determined the eaglet age class using binoculars. We did not climb trees with nests containing either eggs or nestlings younger than 15 days-old, as young eaglets depend on adults for thermoregulation^89^. We planned our climbing approach to avoid subjecting older nestlings to any rain or excessive heat. A team member ascended the nest by rope to install camera-traps (several models of Bushnell camera-traps). Camera-traps were installed 0.5–2 m from nests and fixed using 15-cm-long nails and malleable, 1.65-mm-thick wire. Camera-traps (containing ≥ 16 Gb memory cards and lithium batteries) were programmed to take one photograph every 10 min for 24 h a day. We used 2 or 3 camera-traps per nest to allow multiple angles to view the prey items delivered by adult eagles and minimise data loss from camera-trap failures. Two static camera-traps per nest allowed us to sex the adult eagles by comparing talon morphology, as female harpy eagles have much larger feet and thicker talons^90^. We removed camera-traps after they had photographed a nest for at least 90 days.

### Effects of forest loss on the feeding ecology

**Figure 10:**
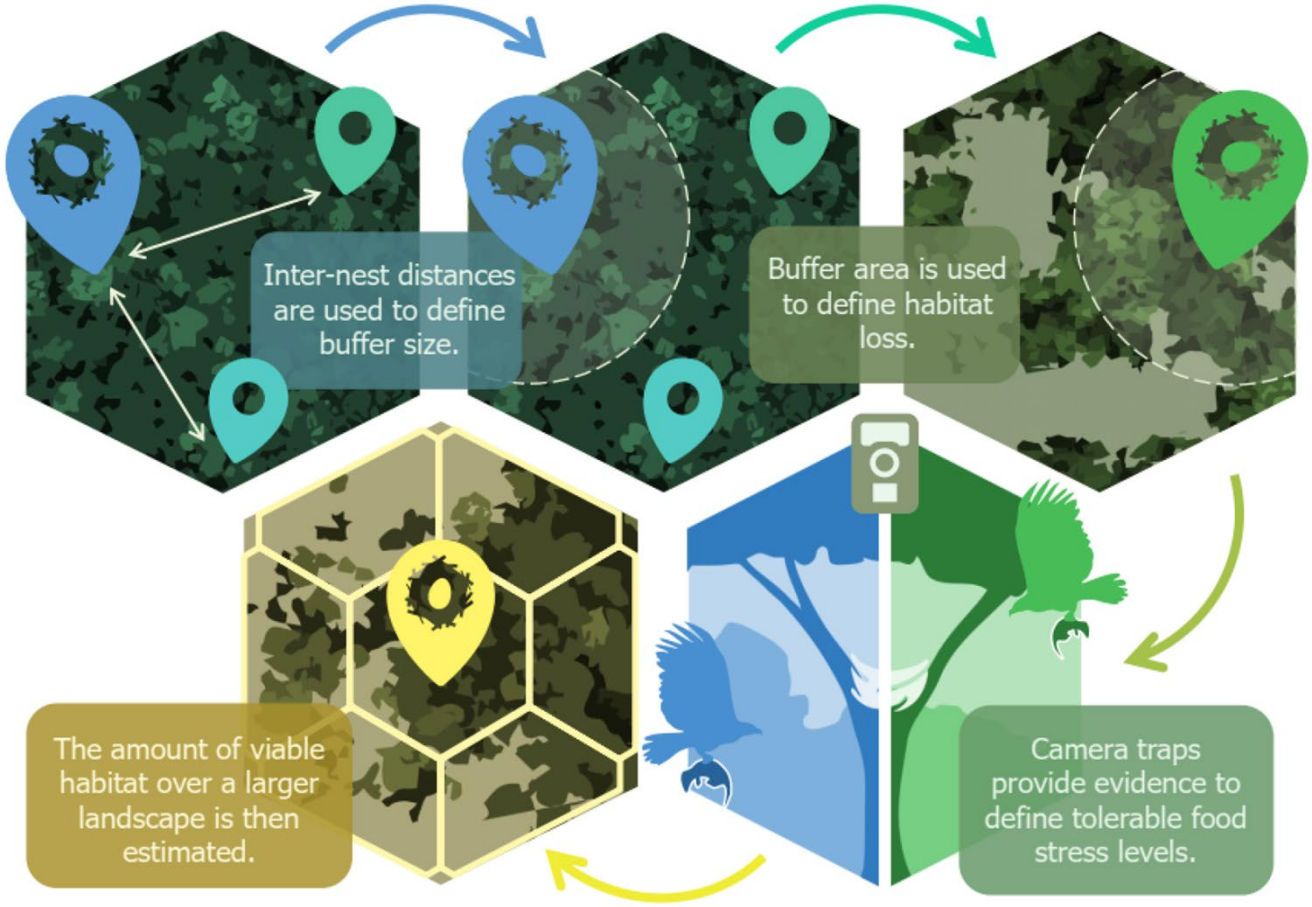
Clockwise flow chart summarizing the steps taken to estimate the effects of habitat loss on harpy eagle population viability. Inter-nest distances are used to define nest-centered landscape buffer sizes. Buffer area is used to obtain data on forest habitat loss. Camera traps provide evidence of tolerable food stress levels. Combining information on tolerable habitat loss and nesting territory size, we can scale up the habitat viability to much larger degraded landscapes (Author: Paula Viana, Corel Draw X8; https://www.coreldraw.com/br).

Addressing complex interactions between landscape ecology and feeding ecology requires a multifaceted approach. The flow chart represented in Fig. 10 shows a step-by-step presentation of our framework, which is also described in detail below.

To define the landscape buffers around each nest, we estimated the nest density using the maximum packaged nest density method (MNPD). The MNPD is based on demarcating a circle around each nest, for which the radius is defined as half the mean distance to the nearest neighbouring nest in a cluster of nests^91^. A constant is then added to fill the interstitial spaces between circles using the equation A = π × r^2^ × 1.158. We used two nest clusters, with six and five nests each, respectively, in two study subregions in which we had comprehensively surveyed the forest (using the reward system) over many years^86^, so we assumed that all harpy eagle nests in those two areas had been detected.

We estimated the percentage of forest area lost within each of those buffers using land cover data available from the MapBiomas Project (collection 5, year 2019; mapbiomas.org). MapBiomas-classified rasters have a georeferenced, 30-m pixel resolution and a general classification accuracy of 97.3% for the Amazon biome. We used Google Earth’s Engine cloud computing platform (code.earthengine.google.com) to access the MapBiomas 2019 collection and extract all pixels corresponding to natural forest formation (ID code 3). We further assessed and added data on recent deforestation that was revealed in post-2019 Google Earth images.

Within the buffers described above, we analysed the effects of habitat loss on prey species composition. In addition to the amount of forest cover, we added distance in meters to the nearest pasture areas as a covariate because even pairs of harpy eagles nesting within forest territories could access open-habitat prey in nearby pastures. We identified prey species from photographs using reference information in the zoological literature^92,93^, and reference collections at the Federal University of Mato Grosso (UFMT), Brazil. We also identified prey skeletal material collected both underneath and inside our study nests.

We defined prey biomass delivery rates by estimating the prey biomass delivered to each nest (using prey data exclusively from camera-traps). Harpy eagles frequently prey on subadult individuals and reduce the weight of large-bodied carcasses to reduce drag during flight^42^. Eagles reduce flight loads (i.e. prey mass) by consuming parts of the carcass. We, therefore, used the following estimated reductions: subadult prey were estimated to represent 66% of adult body mass; for very young prey (because ungulates taken by the eagles are almost exclusively newborns^26^), we estimated that they represented 20% of adult body mass; sloths received a further 33% reduction, because of the large amount of gut foliage content, which represents ~ 30% of the body mass of living sloths^94^. After killing sloths, eagles discard the digestive tract. For dismembered prey, we used the following approximate body mass reductions: 10% (head or viscera), 20% (reduced per arm/leg missing or added per single-member delivered), 50% (lower or upper body missing), and 90% (prehensile tails of Atelinae primates from the genera *Ateles*, *Lagothrix* and *Alouatta*; tails of porcupines, Rodentia: Erethizontidae; tails of lesser anteaters, *Tamandua tetradactyla*) of the total carcass mass^7^. Body masses of these prey species were obtained from the literature^92,95^. When calculating the overall body mass reduction for analyses related to the feeding ecology (rather than biomass delivered to nests), we only reduced whole body mass to account for subadults, rather than including body part reductions. This allowed us to account for the mass of prey parts consumed by adults before carcass delivery at the nest.

We used the forest habitat amount remaining around each nest to determine how much forest loss harpy eagles could tolerate while still exhibiting clear evidence of successful breeding. We defined a hexagonal cell area representing the home range of a harpy eagle pair using the mean distance to the nearest neighbouring nest. Deforestation levels around known active nests surrounded by the highest levels of forest loss—but still successfully raising eaglets—were used to identify hex-cells containing sufficient amounts of forest cover assumed to be suitable for harpy eagles. We defined those below this threshold as unsuitable habitat. We then extended this rationale for the entire 428,800-km^2^ area of the northern portion of the State of Mato Grosso, and calculated the amount of regional-scale habitat suitability both for the first (1985) and last year (2019) of our land-use time series.

### Statistical analyses

We compared prey profiles between the two sexes for several traits such as prey size and Levin’s niche breadth index, using null models^96^. Levin’s index is defined as B_sta_ = B − 1/(n − 1), where B is Levin’s index (B = 1/Σp_j_^2^), p_j_ is the frequency of occurrence of each prey species (or prey item), and n is the total number of prey species^97^. Our null model consisted of the following steps: (1) bootstrapping samples of 25 prey records for both males and females; (2) calculating niche breadth for each sex using a niche breadth measure; (3) creating a pairwise difference in niche breadth between males and females; and (4) determining whether the difference in niche width found between breeders and floaters was larger than expected by chance by comparing differences between two randomly labelled bootstrapped samples (n = 25). We carried out 1000 iterations to calculate niche width differences between sexes, and examined to what extent this departed from chance alone. We selected a sample size of 25 prey items, given that previous work showed this sample to be sufficiently large to adequately represent all prey species brought to active nests at frequencies exceeding 5%^26^. Alpha levels were defined as 5%.

We tested whether the amount of forest loss within buffer areas around each nest affected prey species composition using non-metric multidimensional scaling (NMDS) ordination. We selected the NMDS with the goal of collapsing information from multiple dimensions (in our case, a nest by prey species matrix) into a few axes so that they could be interpreted. To explain the effects of forest loss on harpy eagle feeding ecology, we used the amount of deforestation and the linear distance to the nearest pasture area (in meters). Deforestation likely limits harpy eagle access to canopy prey such as sloths and monkeys, while exotic pastures increase access to ground-dwelling alternative prey such as armadillos.

We also tested the effect of forest loss within nest neighbourhoods on nest-specific prey species richness and Levin’s niche breadth index. We then used the amount of forest loss and distance to nearest pasture as covariates explaining Levin’s niche breadth and prey species richness based on a Generalized Linear Model (GLM). We used a Gaussian error structure and a logit link function^98^.

To determine the impact of forest loss on the feeding ecology of harpy eagles, we used GLMMs with a Gaussian error to explain prey delivery rates both in terms of individual prey items and prey biomass (defined as response variables). We defined feeding rates as the time interval (in days) between consecutive prey deliveries by adults, for which 0 represents two prey items delivered on the same day. We further added the nesting phase (nestling, fledgling and dependent juvenile) as ordinal covariates since these provision intervals slowly increase from 1.8 to 5.12 days per prey item from the nestling to dependent juvenile phases, respectively^99^. We, therefore, used each nest phase per nest to be an independent replica after adding each nest as a random factor in the GLMMs. All analyses were performed using the R coding environment, version 4.0.2^100^.

## Supplementary Information

Supplementary Information.
