# Supplementary material for: Tropical deforestation induces thresholds of reproductive viability and habitat suitability in Earth’s largest eagles
Source: Sci Rep. 2021 Jun 30;11:13048. doi: 10.1038/s41598-021-92372-z (PMC8245467; doi:10.1038/s41598-021-92372-z)
Supplement: Supplementary file 1 — Supplementary Information. [file 41598_2021_92372_MOESM1_ESM.docx]

Formatted for Scientific Reports

**Tropical deforestation induces thresholds of reproductive viability and habitat suitability in Earth’s largest eagles**

Everton B. P. Miranda*^1^, Carlos A. Peres^2^, Vitor Carvalho-Rocha^3^, Bruna V. Miguel^4^, Nickolas Lormand^5^, Niki Huizinga^6^, Charles A. Munn^7^, Tiago B. F. Semedo^8^, Thiago V. Ferreira^9^, João B. Pinho^9^, Vitor Q. Piacentini^10^, Miguel Â. Marini^11^, Colleen T. Downs^1^

*1 – Centre for Functional Biodiversity, School of Life Sciences, University of KwaZulu-Natal, P/Bag X01, Pietermaritzburg, 3209, South Africa*

*2 – School of Environmental Sciences, University of East Anglia, Norwich NR47TJ, UK and Instituto Juruá, Rua Belo Horizonte, 19, Manaus, Brazil*

*3 – School of Environmental Sciences, University of East Anglia, Norwich NR47TJ, UK and Departamento de Ecologia e Zoologia, Universidade Federal de Santa Catarina, Florianópolis, 88040-900, Santa Catarina, Brazil*

*4 – Medicina Veterinária, Universidade Federal de Mato Grosso, Sinop 78550-728, Brazil*

*5 – New Mexico Highlands University, New Mexico, 87701, United States*

*6 – HAS University, 90108, 5200 MA, Netherlands*

*7 – SouthWild, Várzea Grande, Mato Grosso, 78125-048, Brazil*

*8 – Instituto Nacional de Pesquisa do Pantanal (INPP), Museu Paraense Emílio Goeldi (MPEG) - Programa de Capacitação Institucional, Cuiabá, Mato Grosso, 78735-901, Brazil.*

*9 – Programa de Pós-graduação em Ecologia e Conservação da Biodiversidade, Instituto de Biociências, Universidade Federal de Mato Grosso, Cuiabá, Mato Grosso, 78735-901, Brazil*

*10 – Programa de Pós-graduação em Zoologia, Departamento de Biologia e Zoologia, Instituto de Biociências, Universidade Federal de Mato Grosso, Cuiabá, Mato Grosso, 78735-901, Brazil*

*11 – Departamento de Zoologia, IB, Universidade de Brasília, Brasília, 70910-900, Distrito Federal, Brazil*

***Corresponding author:** [mirandaebp@gmail.com](mailto:mirandaebp@gmail.com), phone +55 65 98153550

ORCID: <https://orcid.org/0000-0003-2198-4742>

**Other emails and ORCIDs:**

c.peres@uea.ac.uk; ORCID: <http://orcid.org/0000-0002-1588-8765>; [downs@ukzn.ac.za](mailto:downs@ukzn.ac.za); ORCID: <http://orcid.org/0000-0001-8334-1510>;

[marini@unb.br](mailto:marini@unb.br); ORCID: http://orcid.org/0000-0002-7300-7321

**Running header:** Harpy eagle feeding ecology and landscape degradation

**SI Table 1.** General problems faced with camera trap use in nests. The 37 camera-traps installed at harpy eagle nests had several issues. Configurations reset in 8.1% of cameras, producing photographs every couple of seconds with the wrong dating and timing. Epiphytic foliage grew in front of 5.4% of camera-traps, obscuring prey view and causing the camera-traps to trigger without the presence of a harpy eagle. Poor positioning occurred in 16.2% of all camera-traps and created issues for prey identification. The nest section being monitored by a camera-trap fell naturally in 5.4% of nests. Nails hammered shallowly in the bark caused the camera-trap to dislodge losing the view of the nest, or even to fall, in 8.1% of camera-traps. Finally, camera-traps completely failed in 29.7% of all occasions, which was a high toll considering that none of the other issues are necessarily fatal to sampling. All camera-traps suffered minor damage from eaglets playing with them, but on no occasion it was an issue for sampling prey. No adults were seen interacting with the camera-traps. First column shows nests, the second shows the camera trap number (2-3 per nest), CD column has the camera-days, and Photos represent the number of photos per camera.

| **Nest** | **Cam** | **CD** | **Photos** | **Configurations** | **Epiphytic leaves** | **Bad positioning** | **Nest section fell** | **Nails** | **Camera failed** |
| --- | --- | --- | --- | --- | --- | --- | --- | --- | --- |
| 1 | I | 75 | 318 |  |  |  |  |  |  |
|  | II | 75 | 76 |  |  | x |  | x |  |
|  | I | 79 | 190 |  |  |  |  |  |  |
|  | II | 1 | 4 |  |  |  |  |  | x |
| 2 | I | 52 | 3365 | x |  |  | x |  |  |
|  | II | 134 | 498 |  |  |  |  |  |  |
| 3 | I | 107 | 267 |  |  |  |  |  |  |
|  | II | 76 | 1006 |  |  |  |  |  |  |
| 4 | I | 92 | 6987 |  | x |  |  |  |  |
|  | II | 101 | 1106 |  |  |  |  |  |  |
| 5 | I | 5 | 956 |  |  | x |  |  |  |
|  | II | 1 | 2 |  |  |  |  |  | x |
|  | III | 10 | 1116 |  |  | x |  |  |  |
|  | I | 1 | 7 |  |  |  |  |  | x |
|  | II | 5 | 45 |  |  |  |  |  | x |
| 6 | I | 4 | 44 |  |  | x |  |  | x |
|  | II | 2 | 2 |  |  | x |  |  | x |
|  | I | 166 | 778 |  |  | x |  |  |  |
|  | II | - | - |  |  |  |  |  | x |
| 7 | I | 104 | 864 |  |  |  |  |  |  |
|  | II | 104 | 1230 |  |  |  |  | x |  |
| 8 | I | 119 | 793 |  |  |  |  |  |  |
|  | II | - | - |  |  |  |  |  | x |
| 9 | I | 27 | 438 |  |  |  |  |  |  |
|  | II | 98 | 1012 |  |  |  | x |  |  |
| 10 | I | 53 | 209 |  |  |  |  |  |  |
|  | II | - | - |  |  |  |  |  | x |
|  | I | 11 | 92 |  |  |  |  |  | x |
|  | II | 39 | 354 |  |  |  |  |  |  |
| 11 | I | 81 | 391 |  |  |  |  |  |  |
|  | II | 79 | 389 |  |  |  |  |  |  |
| 12 | I | 109 | 1558 |  |  |  |  |  |  |
|  | II | 73 | 1753 |  |  |  |  |  |  |
| 13 | I | 52 | 1187 | x | x |  |  |  |  |
|  | II | - | - |  |  |  |  |  | x |
| 14 | I | 31 | 331 |  |  |  |  | x |  |
|  | II | 26 | 10059 | x |  |  |  |  |  |
